# Photoplethysmography-Based Respiratory Rate Estimation Algorithm for Health Monitoring Applications

**DOI:** 10.1007/s40846-022-00700-z

**Published:** 2022-04-07

**Authors:** Talha Iqbal, Adnan Elahi, Sandra Ganly, William Wijns, Atif Shahzad

**Affiliations:** 1 Smart Sensor Lab, Lambe Institute of Translational Research, College of Medicine, Nursing Health Sciences, National University of Ireland, Galway, H91 TK33 Ireland; 2 Department of Electrical Engineering, National University of Ireland, Galway, H91 TK33 Ireland; 3 CÚRAM Center for Research in Medical Devices, Galway, H91 W2TY Ireland; 4 Centre for Systems Modelling and Quantitative Biomedicine (SMQB), University of Birmingham, Birmingham, B15 2TT UK

**Keywords:** Photoplethysmography, Respiratory rate, Adaptive estimation, Wearable sensors, Health monitoring, Algorithms

## Abstract

**Purpose:**

Respiratory rate can provide auxiliary information on the physiological changes within the human body, such as physical and emotional stress. In a clinical setup, the abnormal respiratory rate can be indicative of the deterioration of the patient's condition. Most of the existing algorithms for the estimation of respiratory rate using photoplethysmography (PPG) are sensitive to external noise and may require the selection of certain algorithm-specific parameters, through the trial-and-error method.

**Methods:**

This paper proposes a new algorithm to estimate the respiratory rate using a photoplethysmography sensor signal for health monitoring. The algorithm is resistant to signal loss and can handle low-quality signals from the sensor. It combines selective windowing, preprocessing and signal conditioning, modified Welch filtering and postprocessing to achieve high accuracy and robustness to noise.

**Results:**

The Mean Absolute Error and the Root Mean Square Error of the proposed algorithm, with the optimal signal window size, are determined to be 2.05 breaths count per minute and 2.47 breaths count per minute, respectively, when tested on a publicly available dataset. These results present a significant improvement in accuracy over previously reported methods. The proposed algorithm achieved comparable results to the existing algorithms in the literature on the BIDMC dataset (containing data of 53 subjects, each recorded for 8 min) for other signal window sizes.

**Conclusion:**

The results endorse that integration of the proposed algorithm to a commercially available pulse oximetry device would expand its functionality from the measurement of oxygen saturation level and heart rate to the continuous measurement of the respiratory rate with good efficiency at home and in a clinical setting.

**Supplementary Information:**

The online version contains supplementary material available at 10.1007/s40846-022-00700-z.

## Introduction

In recent years, respiratory rate (RespR), blood pressure (BP) and heart rate (HR) monitoring are considered essential for continuous and primary assessment of the patient’s well-being [1]. The inhalation and exhalation process can increase or decrease the blood flow within the body. Therefore, respiratory rate can be determined by measuring the changes in the heartbeats or blood flow [2–5]. There is significant evidence in the literature to suggest that irregular respiration is an imperative indicator of some serious illness [6–8]. The normal range of respiratory rate for children (1–5 years) is above 24 and less than 40 breaths per min while above 5 years, the normal range is between 12 and 25 breaths per minute. Any deviation from the normal range is an indicator of respiratory distress and requires instant clinical intervention [9, 10]. According to the World Health Organisation (WHO), elevated respiratory rate is observed in the cases of chronic obstructive pulmonary disease, asthma, hypoxia, and pneumonia [11, 12].

In hospitals, respiratory rate is monitored using thoracic/abdominal plethysmography belts, oral/nasal pressure transducers, capnography, and transthoracic impedance pneumography [13, 14]. However, these devices are not as user-friendly as mobile wearable devices. Most smartwatches use a photoplethysmography signal to extract only the heart rate even though the PPG signal can also be used to extract RespR [15, 16]. While algorithms have been proposed in the literature to extract respiratory rates from PPG signals, each algorithm has certain limitations. Estimation of respiratory rate from a PPG signal can be achieved by using digital signal processing (DSP) techniques. Among these techniques, digital filters are commonly used to remove noise and extract variables of interest from the raw signals. However, the performance of DSP filters is highly dependent on the cut off frequency of the filter. Other techniques include analytical methods, which are very sensitive to noise and result in very poor respiratory rate detection in presence of motion artefacts. Time–frequency analysis-based methods such as Wavelet transform addresses most of the common problems of filtering and analytical methods. It is less sensitive to noise and motion artefacts but requires the selection of more than one parameter, such as mother wavelet function and the total number of decomposition levels, which in practice are unknown [17, 18]. A list of various categories of respiratory rate estimation methods and their limitations is provided in Table 1.

**Table 1:** Respiratory rate estimation methods and their limitations

Methods		Limitations
Digital method	Digital technique (FFT, Welch, Notch) [23, 24]	Highly dependent on the selection of cut-off frequencies
Wavelet methods	Wavelet transforms [25, 26]	Requires the selection of more than one parameter such as the mother wavelet function and the total number of decomposition levels
	Smart fusion [20]	
Adaptive estimations	Adaptive respiratory rate estimators [23, 27]	Very sensitive to noise and results in very poor respiratory rate estimation if there are any motion artefacts in the signal
	Empirical mode decomposition (EMD) [28, 29]	
Analytical methods	Autoregression [30, 31]	Often requires a relatively long time to converge and give an accurate estimation of respiratory rate
	Artificial neural networks [32]	
	Principal component analysis (PCA) [33]	
	Complex demodulation [34],	
	Independent component analysis (ICA) [35]	

One of the major challenges in respiratory rate estimation from PPG signal is respiratory induced amplitude variation [19]. During the inhale cycle, the intra-thoracic pressure changes cause decreased stroke volume of the left ventricle, which leads to a smaller PPG amplitude. Similarly, during expiration, the left ventricle stroke volume increases, which results in increased pulse amplitude. In the literature, a variety of methods have been proposed for the estimation of RespR from a PPG signal. Liu et al. [15] have highlighted the merits and demerits of different respiratory rate estimation algorithms. Another key challenge in developing a respiratory rate extraction algorithm is the estimation of optimal window size for the segmentation of the signal. A shorter time window provides high resolution, low computational cost, and better real-time performance. In contrast, a longer window size provides better estimation accuracy [20]. The proposed algorithm is developed considering all the major limitations including noise and poor signal quality, the effect of window size, and cut-off frequencies of the filters.

For evaluation of the proposed algorithm, the estimated respiratory rate is compared with the reference respiration data provided in the publicly available dataset called BIDMC dataset. The dataset is available at PhysioNet [21] while for filtering and signal process an open-source toolkit Heartpy [22] with some modification was used. The performance of the proposed algorithm was benchmarked using accuracy assessment metrics against published results of existing algorithms. The rest of the paper is organized as follows: Sect. 2 provides an overview of the methods for preprocessing, signal processing and post-processing steps of the proposed algorithm; Sect. 3 presents a brief discussion of the BIDMC dataset and evaluation metrics used to validate the proposed algorithm. Section 4 shows the results and discussion, while conclusions are provided in Sect. 5.

## Proposed Algorithm

**Fig. 1:**
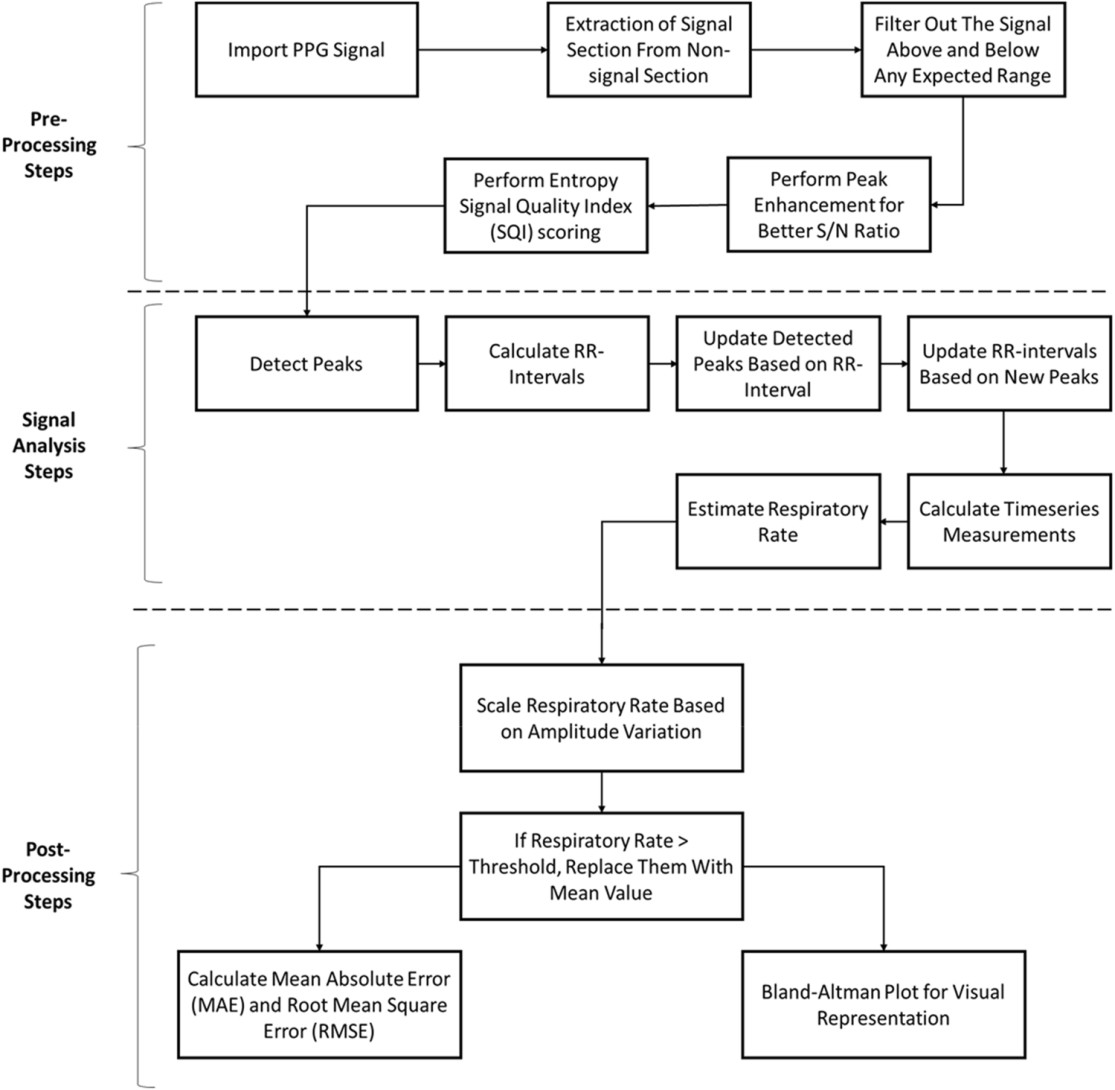
Pre-processing, Signal analysis and Post-processing steps of the respiratory rate estimation algorithm

Figure 1 shows the pre-processing, signal analysis and post-processing steps of the proposed respiratory rate estimation algorithm.

### Pre-processing Steps

For pre-recorded datasets, there is one additional step of signal interpolation included in the pre-processing stage, as explained below. All the other steps are shown in Fig. 1 and the same for pre-recorded as well as real-time PPG signals.

#### Signal Interpolation

The first step in the pre-processing stage for a pre-recorded dataset is to extract the raw PPG signal values from the dataset and reference respiratory rate values. In the literature, several techniques have been proposed to address the problem of a missing signal [36, 37]. The simplest and easiest way to tackle this problem is to remove the signal points with missing values. However, it is recommended to interpolate for missing data as eliminating or inserting zeros causes a complete loss of the data (information or signal) [38]. This loss of data might play an important role in deriving conclusions or in determining any statistical outcome. Thus, replacing the missing signal, generally marked as NaN (not a number), by the mean value of two neighbouring signal samples, might not affect the overall signal behaviour and derived conclusions/results can be considered valid [39].

#### Digital Filtering

**Fig. 2:**
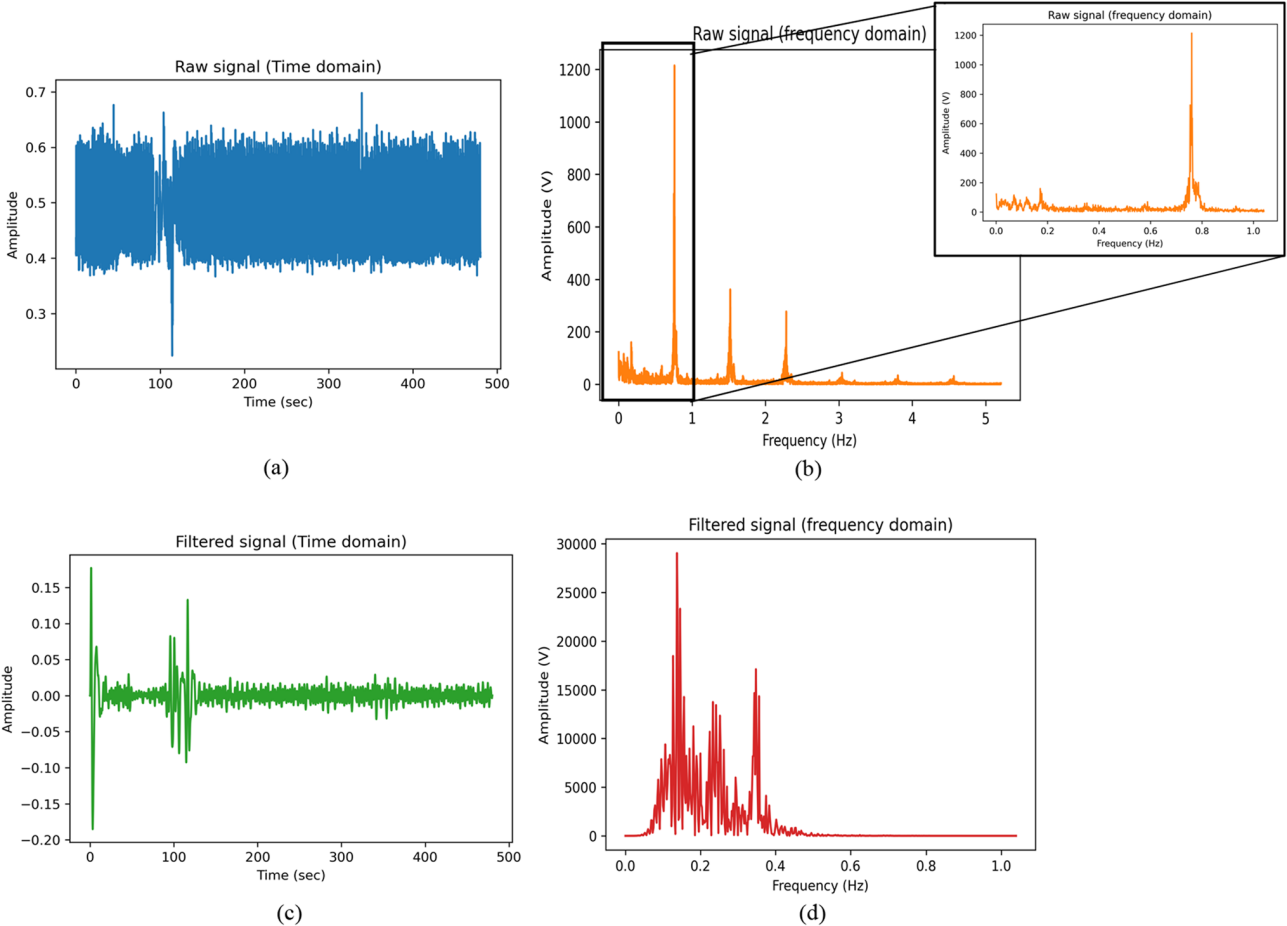
Extraction of respiratory rate signal from raw PPG signal. **a** shows the raw PPG signal imported from the dataset **b** is the frequency domin signal of the same raw PPG signal (clipped to frequency = 5 Hz) **c** illustrates the filtered signal passed through band pass butterworth filter with cutoff frequency of 0.1–0.4 Hz while **d** is the frequency domain representation of the filtered signal. Note that only the frequencies between 0.1 and 0.4 are passed and all other are blocked (showing flat line)

The raw PPG signal has information on heart rate and respiratory rate as well as noise. The digital filtering method is used to remove the noise and extract the relevant information. The raw PPG signal is passed through a bandpass Butterworth filter to allow only the frequencies within the range of minimum and maximum respiratory rate (i.e., 0.1–0.4 Hz or 6–24 breaths per minute), as shown in Fig. 2.

#### Peak Enhancement

To enhance the signal to noise ratio and to get better detection of the peaks, the algorithm performs peak enhancement using the peak enhancement algorithm. This is a crucial step as all the further calculations will be dependent on the detected peak. Thus, accurate detection of peaks will play a significant role in this algorithm. This function makes the higher peaks more dominant while suppressing the smaller peaks, for better detection of the peaks. This function scales the signal to the specified lower and upper range. The formula is as in Eq. (1):

1 $${P}_{enh}= rb * ((x-min(x))/range(x)) + l\_lt$$

where ${P}_{enh}$ is peak enhancement, $x$ is the raw signal, $rb$ is the range of upper limit and $l\_lt$ (lower limits), given by the user (by default it is set at 1024 and 0, respectively). The $range(x)$ is a valued range calculated by subtracting the maximum value of the analysed signal from the minimum value.

#### Outlier Detection

Most of the time, PPG devices have loose contact with the body causing abrupt changes, possibly due to sudden moves of the finger or by other unknown reasons, in the raw signal. Outlier detection function is required to eliminate these baseline abrupt changes. These changes could not be completely removed by general digital filters as they contain wide-band frequency components. In the developed algorithm, the outlier can be removed using the Hampel filtering technique [40, 41].

The goal of the Hampel filter is to identify and replace outliers in each window analysed. It uses a sliding window of configurable width to go over the signal. The median and the standard deviation is calculated for each window, of *x* seconds, and expressed as the median absolute deviation (MAD). For each sample of *x*, the algorithm computes the median of a window composed of the sample and its six surrounding samples (if window size = 6), three per side. If a sample differs more than the median + three standard deviations, it is replaced with the median. As the algorithm uses 6 neighbouring samples (data point + 3 per side) to cover most of the signal, only the last three sample/data points are left at the end.

**Fig. 3:**
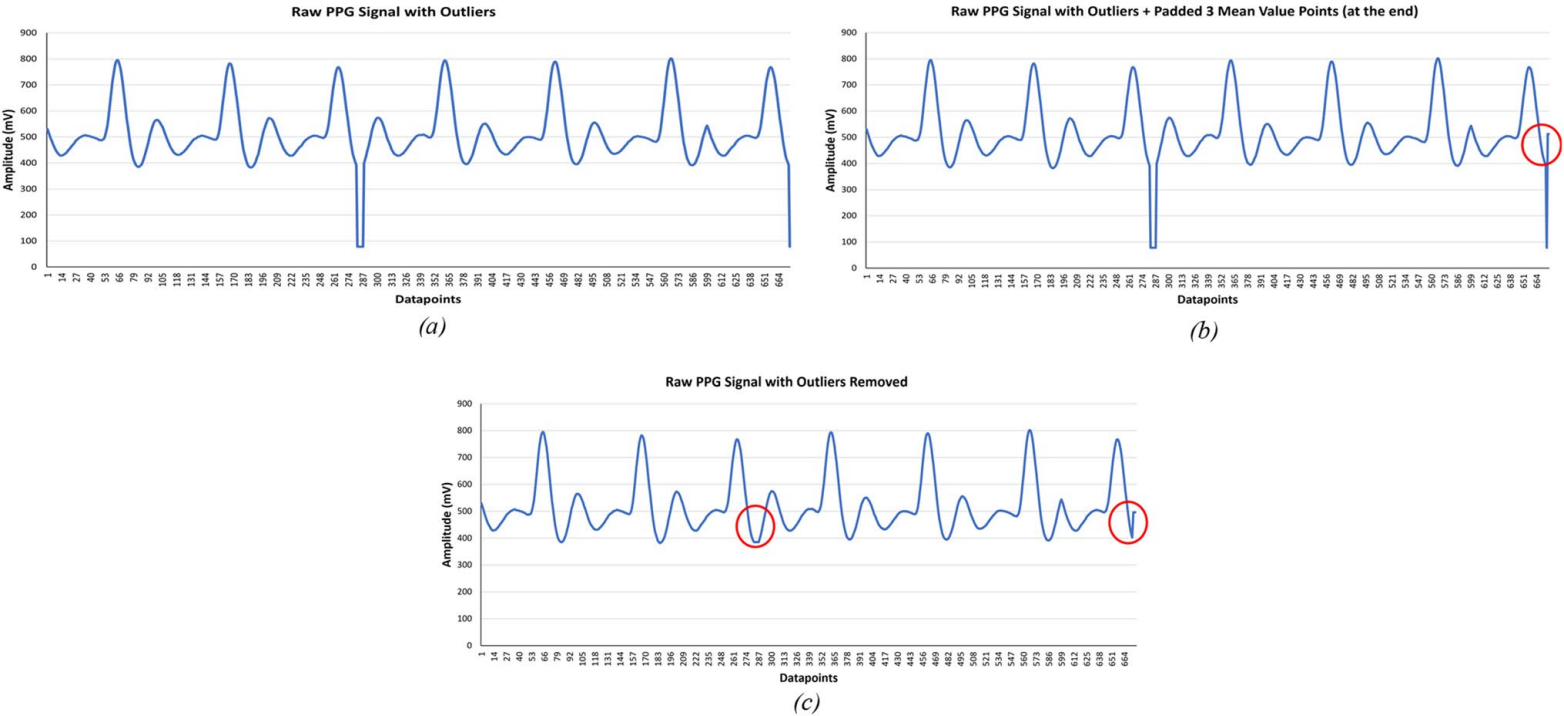
Outlier removal using Hampel filter with window size = 6

To ensure those points are included for outlier detection, three points (mean value of the signal) are padded at the end of the signal. The sliding window is moved until the last sample of the signal. Figure 3 shows a sample example of how outlier removal works. Figure 3a exemplifies an outlier present in a PPG signal, Fig. 3b demonstrates padding of additional three-points in a PPG signal while Fig. 3c shows the output of the Hampel filter and interpolated PPG signal after the removal of the outlier.

#### Entropy-Based Signal Quality Index (ESQI)

One of the objectives of the proposed algorithm is to accurately extract the respiratory rate even from a low-quality signal. Entropy Signal Quality Index (ESQI) scoring was proposed by Selvaraj et al. [42]. It quantifies the difference of the probability distribution (PDF) of raw signal from the uniform (normal) distribution and provides a measure of uncertainty present in the analysed signal [43]. The equation to calculate ESQI is as:

2 $${E}_{SQI}= - \sum_{i=1}^{N}{x\left[i\right]}^{2} {log}_{e}\left({x\left[i\right]}^{2}\right)$$

In Eq. 2, $x$ is the raw PPG signal and *N* is the number of points within the analysed raw PPG signal. The signal quality assessment revealed that for some portion of the signal, ${x\left[i\right]}^{2} \to 0$(no or fewer fluctuations) then ${log}_{e}\left({x\left[i\right]}^{2}\right)\to nan$. Thus, the ESQI value returns as undefined. The algorithm skips the further computation for respiratory rate estimation if the ESQI is undefined $(nan)$.

### Signal Analysis and Respiratory Rate Estimation

#### Peak Detection

**Fig. 4:**
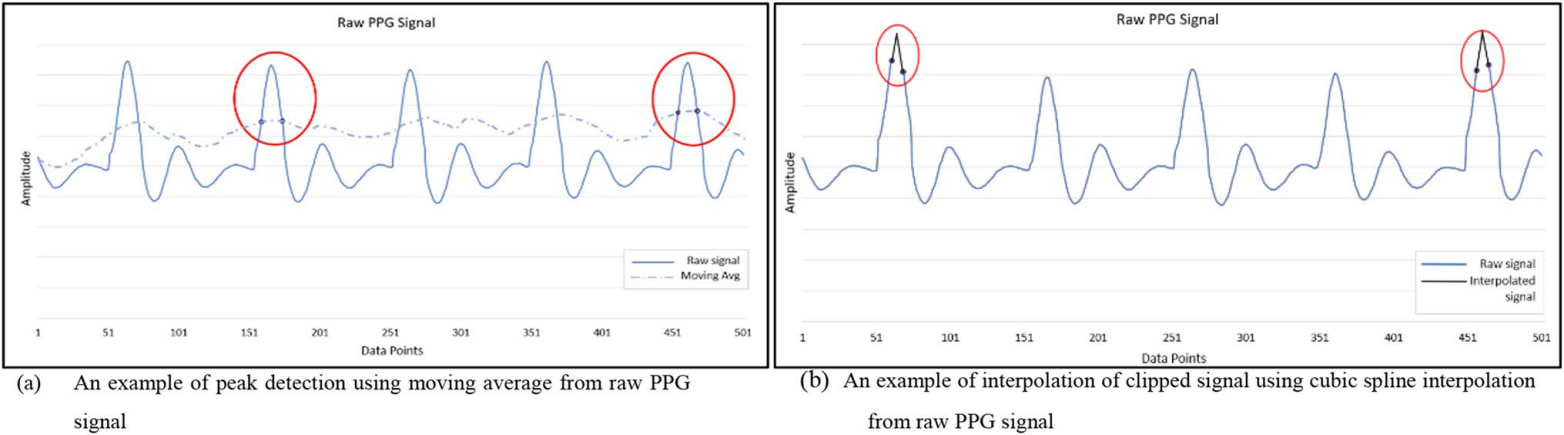
Peak detection and interpolation of the clipped signal

The next step is to analyse the signal. The algorithm detects peaks within the peak enhanced signal. This is a crucial step as all the further calculations will be dependent on the detected peak. Once peaks are detected, the algorithm calculates the systolic peak interval or RR interval. Peak detection can be done by using a moving average. An intersection threshold is made, and Region of Interest (ROI) is selected between two intersection points where the signal amplitude is the highest, as shown in Fig. 4a. If the raw PGG signal had a clipped signal, the algorithm uses cubic spline interpolation to interpolate the missing signal before peak detection which is shown in Fig. 4b. The red circles in Fig. 4a marks ROI and two intersecting points are used to determine the peak while in Fig. 4b they show the clipped signal (in blue) and cubic spline interpolated signal (in black).

#### Respiratory Rate Estimation

The peak detection algorithm may also detect some false peaks [44, 45]. The proposed algorithm ensures that the algorithm detects only true peaks by removing the false peaks. The peak is considered to be a false peak if the R–R interval between two adjacent R-peaks is less than 30% of the mean R–R interval of the analysed signal, as mentioned in [22] and any false peak is dropped. The R–R intervals are calculated again after dropping any false peaks. The new values of the RR interval are then used to calculate different time-series measurements. These measurements include heart rate in beats per minute (BPM), RR-intervals or inter-beat interval (IBI) and estimated respiratory rate in breaths per min. The respiratory rate is calculated using Welch’s method [46, 47]. Welch’s method divides the inter-beat intervals (signal) into overlapping segments and computes a modified periodogram for each segment. Then an average of all periodograms along with an array of frequencies are returned at the output. The respiratory rate is the maximum frequency within the frequency band (Hz) that is, where PSD is maximum. To calculate the final respiratory rate (RespR) the frequency is multiplied by 60 to convert the respiratory frequency band from Hz to breaths per minute. Mathematically it is represented as,

3 $$\mathrm{Respiration\, rate}=\mathrm{f}\times \mathit{arg}\underset{i}{\mathit{max}}P\left(i\right)\times 60$$

In Eq. 3, f is the frequency while P is power spectral density calculated by the welch method. Table 2 describes the parameters initialization values used in the proposed algorithm.

**Table 2:** Welch filter parameters for determining respiratory rate

S. No.	Parameter	Value/Method
1	Sampling frequency	125
2	Window	Hann Window
3	Number of overlapping points	50%
4	Length of FFT	Length of data
5	Scaling	Density
6	Averaging periodogram	Mean

### Post-processing Steps

Usually, the PPG waveform varies in-synchrony with the respiratory cycle [48]. During the inhale cycle, the intra-thoracic pressure changes causing decreased stroke volume of the left ventricle, which leads to a smaller amplitude (PPG) pulse. Similarly, during expiration, the left ventricle stroke volume increases, which increases the pulse amplitude. This phenomenon is known as amplitude modulation of cardiac synchronous pulsatile waveform or respiratory induced amplitude variation [19].

As in the prior stages, the proposed algorithm does not account for amplitude variation, due to active filtering and peak enhancement steps, which is essential for accurate peak detection; In the post-processing stage, the estimated respiratory rate is scaled based on the variation (changes) in the amplitude of the PPG signal. The scaling is essential as the proposed (and most of the existing algorithms) does not account for amplitude variation in the pre-processing stage. Thus, a separate scaling is applied to the estimated respiratory rate. This scaling factor depends on the range (difference between maximum and minimum value) of the signal and defined window size (of $x$ seconds).

The proposed scaling method is generalisable and can work for a variety of PPG datasets. Nevertheless, there might be scenarios where some fine-tuning may be required for better estimation. As the last step, the algorithm makes sure the estimated values of the respiratory rate are within a specific threshold and do not exceed the maximum physiologically possible breathing rate.

## Validation of Proposed Algorithm

To validate the proposed algorithm and assess its performance, a publicly available dataset, known as Berth Israel Deaconess Medical Centre (BIDMC) dataset, was used. The proposed algorithm was applied to the PPG data in the BIDMC dataset for estimation of respiratory rate and the performance was benchmarked against various existing algorithms using the most common performance evaluation metrics.

### BIDMC Dataset Overview

The dataset consists of the electrocardiogram (ECG), photoplethysmogram (PPG) and impedance pneumogram respiratory signals of patients in the intensive care unit at Berth Israel Deaconess Medical Centre (BIDMC), Boston, USA [21, 49]. The presented dataset was proposed to evaluate the performance of any newly developed respiratory rate algorithm and reflect its potential usability in a real-world critical care environment. Table 3 shows the key statistical features of the BIDMC dataset. The dataset is comprised of 53 patients’ recordings, each of 8-min duration, containing:

- Physiological signals; were sampled at 125 Hz.
- Physiological parameters; such as respiratory rate, blood oxygen saturation levels and heart rate sampled at 1 Hz.
- Age and gender; are fixed parameters.
- Manually annotated individual breaths, annotated independently by two researchers.

**Table 3:** Key statistical features of the respiratory rate in BIDMC dataset (unit = breaths per minute)

N	Validated	53	
	Outlier	2 (Subject 13 and 33)	
		With outlier	Without outlier
Mean		17.42	17.63
Median		17.89	17.89
Standard Deviation		3.22	2.62
Variance		10.39	6.86
Minimum		3.71	10.47
Maximum		24.67	24.67

### Performance Evaluation Metrics

For evaluation of the developed algorithm, Mean Absolute Error (MAE) and Root Mean Square Error (RMSE) metrics were used, and the performance of the proposed algorithm was compared with existing algorithms.

- The mean absolute error (MAE) is an average measure of difference (error) between the reference value and the algorithm’s estimated value of that observation. Mathematically, MAE is calculated using Eq. 4 and is as follows:
  4 $$\mathrm{MAE}=\frac{1}{N}{\sum }_{i=1}^{N}\left|{x}_{i}-\widehat{{x}_{\mathrm{i}}}\right|$$
  where ${x}_{i}$ is reference value and $\widehat{{x}_{i}}$ is an estimated value of the signal and N is the total number of samples in the signal.
- The root means square error (RMSE) is a square root of the mean of the square of estimation error. The RMSE shows the standard deviation of the estimation error and is considered a good measure of accuracy. Equation 5 illustrates RMSE mathematically.
  5 $$RMSE=\sqrt{\frac{1}{N}{\sum }_{i=1}^{N}{\left|{x}_{i}-\widehat{{x}_{i}}\right|}^{2}}$$

where ${x}_{i}$ is reference value and $\widehat{{x}_{i}}$ is an estimated value of the signal and N is the total number of samples in the signal.

In Eqs. 4 and 5, the *reference value* denotes the real respiratory rate reported in the BIDMC dataset while the *estimated value* denotes the calculated respiratory rate.

## Results and Discussion

To estimate the respiratory rate and perform estimation error analysis, data of 51 patients in the BIDMC dataset were used, discarding the two outliers mentioned in Table 3. The two patients that are excluded in this study are patient 13 and patient 33. Reference respiratory rate values of patient 13 are missing in the dataset while the raw data of patient 33 are too distorted to extract any meaningful information. The respiratory rate was calculated using window sizes of 10, 20, 30, 45, 60, 90, and 120 s, defined at pre-processing step. For comparison of the developed algorithm with other state-of-the-art algorithms [20, 49–51], a window size of 32 and 64 s was also used to estimate the respiratory rate to match the window sizes with the previously published algorithms.

The smaller window size yields less processing and computation time, but it can give inaccurate readings of respiratory rate. While using a larger window size, the overall accuracy of the estimation can be improved but it is difficult to estimate the lowest detectable respiratory rate [20]. Thus, a careful trade-off is needed while selecting any specific window size for the analysis.

### Performance Evaluation

The proposed algorithm was able to estimate the respiratory rate accurately for all the subjects within the BIDMC dataset excluding subject 18, see supplementary tables S1 and S2. Figure 4 shows the error in the estimation of respiratory rate using different window sizes with and without Entropy-based signal quality (ESQI) assessment. ESQI is generally used in off-line data processing to select good quality signals from raw data, and it improves estimation accuracy, as the poor-quality data is rejected. However, ESQI will result in loss of data in online/real-time applications and may not be applicable in many cases. In this study, ESQI is performed to assess the estimation result of the proposed algorithm and determine whether the proposed algorithm can produce acceptable results without ESQI or not. The MAE and RMSE values (in terms of breaths counts per minute) are close to each other endorsing the claim of accurate estimation of respiratory rate even using a low-quality signal.

**Fig. 5:**
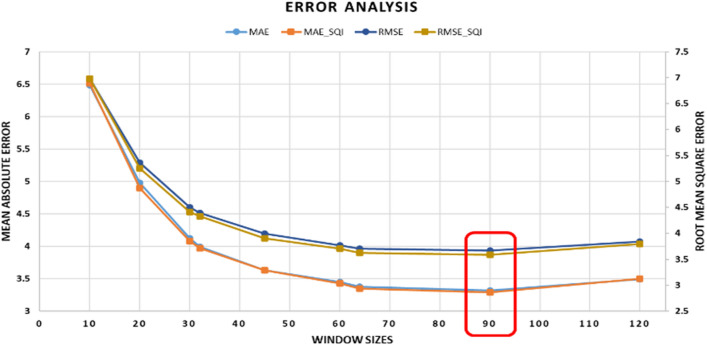
Error analysis of estimated respiratory rate (breaths count per minute) using different window sizes, with and without ESQI

From Fig. 5, it can also be noticed that the minimum mean absolute error (MAE) and root mean square error (RMSE) is achieved by using a window size of 90 s for all the subjects. The error rate continues to decrease till window size = 90 s (highlighted by the red box) and increases for a window size of 120 s.

### Selection of Best Window Size

The best window size for respiratory rate estimation varies subject to subject. In the real-world scenario, the user can calibrate the respiratory rate monitoring device beforehand by taking regular breaths and manually entering it into the device. The device will then compare the estimated respiratory rate with the manually entered value and determine the suitable window size for respiratory rate estimation.

When MAE and RMSE are calculated using the best window size for each subject, the overall error decreases from 3.32 (breaths count per minute) and 3.67 (breaths count per minute) to 2.15 (breaths count per minute) and 2.56 (breaths count per minute) (without any signal quality assessment) while from 3.29 (breaths count per minute) and 3.59 (breaths count per minute) to 2.05 (breaths count per minute) and 2.47 (breaths count per minute) (with ESQI assessment), respectively, as shown in Table 4. This improvement results in an over 35% reduction in mean estimation error.

**Table 4:** Error in respiratory rate estimation using 90 s and best-suited window sizes (unit for MAE and RMSE = breath counts per minute)

Metrics (without E_SQI_ criteria)			Metrics (with E_SQI_ criteria)		
Window	90 s	Best suited	Window	90 s	Best suited
MAE	3.32	2.15	MAE	3.29	2.05
RMSE	3.67	2.56	RMSE	3.59	2.47

### Comparison with State-of-the-Art Respiratory Rate Estimation Algorithms

The performance of the proposed algorithm was compared with Karlen et al. [20], Pimentel et al. [49], Nilsson et al. [50] and Fleming et al. [51]. These algorithms are representative of key studies that are either state-of-the-art or being considered benchmark investigations. Table 5 shows the values of MAE for each algorithm using a window size of 32 and 64 s. The proposed algorithm gives compatible accuracy to the existing algorithms with the MAE of 3.97 (breaths count per minute) and 3.35 (breaths count per minute) for each window, respectively. The algorithm also gives the lowest MAE value i.e., 2.05 if the best window size for each subject is used.

**Table 5:** Comparison of proposed respiratory rate estimation algorithm: Mean Absolute Error (MAE) and Window Sizes

Algorithm	MAE (breaths count per minute)	Window size
Karlen et al*.* [20]	5.80	32
Pimentel et al*.* [49]	4.00	
Nilsson et al*.* [50]	5.40	
Fleming et al*.* [51]	5.20	
Proposed	**3.97**	
Karlen et al*.* [20]	5.70	64
Pimentel et al*.* [49]	2.70	
Nilsson et al*.* [50]	4.60	
Fleming et al*.* [51]	5.50	
Proposed	**3.35**	
Proposed	**2.05**	Best window size^a^

^a^Calculation is done using best window size for each subject; see Table S2 (in supplementary file)

## Conclusion

In this paper, we proposed an algorithm to extract the respiratory rate from a PPG signal. The algorithm is based on three steps that are pre-processing, signal analysis, and post-processing. In the pre-processing stage, the signal is analysed for required signal extraction, filtration of the signal above and below the expected range, and peak enhancement for increasing the signal to noise ratio. In the signal analysis stage, peak detection, peak to peak interval, error in peak detection and correction, updated peak to peak interval, calculation of different time-series measurements and estimation of respiratory rate is done. As the amplitude of the PPG signal is affected by respiratory rate, in the final stage, scaling is performed based on amplitude variation.

For the validation of the proposed algorithm, we used the BIDMC signal set and calculated the mean absolute error and root mean squared error. One of the aims of this study was to determine the impact of different window sizes on the calculation of respiratory rate in real-time. The results in Fig. 4 suggest that a window size of 90 s is best for estimation of respiratory rate using the BIDMC signal set, as it gives minimum MAE and RMSE values. The best window size to estimate the respiratory rate differs from person to person. If the best window size for each subject is used for the error analysis, then the maximum MAE and RMSE of our algorithm decrease to 2.05 (breaths count per minute) and 2.47 (breaths count per minute), respectively (see Table 4).

In the future, the scaling technique will be improved which will eventually improve the estimation accuracy furthermore. The scaling value is the only hyperparameter that might need to be determined empirically. The default method of scaling does work for most of the PPG data but may require improvement in some cases. To solve this problem, the algorithm needs to be evaluated on different datasets to determine a more generalizable scaling value to estimate the respiratory rate accurately.

The developed algorithm can estimate the respiratory rate from the PPG signal collected through a pulse oximeter to provide a simple, cheap, and signal-sensor solution. Integration of the proposed algorithm to a commercially available pulse oximetry device would expand its functionality from the measurement of oxygen saturation level (SpO2) and heart rate to the continuous measurement of the respiratory rate with great efficiency in the clinical setting as well as in the ambulatory home-based environment.

### Electronic supplementary material

Below is the link to the electronic supplementary material.

Supplementary file1 (PNG 152 kb)

Supplementary file2 (PNG 153 kb)

Supplementary file3 (PNG 154 kb)

Supplementary file4 (PNG 147 kb)

Supplementary file5 (PNG 174 kb)

Supplementary file6 (PNG 152 kb)

Supplementary file7 (PNG 152 kb)

Supplementary file8 (PNG 100 kb)

Supplementary file9 (PNG 136 kb)

Supplementary file10 (PDF 340 kb)

Supplementary file11 (PDF 339 kb)

## Appendix

In the supplementary files, Fig. S1 to Fig. S9 shows the Bland–Altman plot of all the subjects using a window size of 10, 20, 30, 32, 45, 60, 64, 120 and best window in seconds. According to the United States Food and Drug Administration (FDA), repeated measurements through a device must lie within the allowed 3Ϭ (± 3* standard deviation) range to be classified as a Class II medical device [52]. In the proposed case, the bias values of all the Bland–Altman plots in the supplementary files are near zero while 95% of the data lies within the limits of agreement (± 1.96 * standard deviation), Tabulated in Table

**Table A1:** Bland–Altman plot: bias values along with upper and lower limits of agreement

Window size	Bias value	Standard deviation of bias	Limit of agreement	
			Lower	Upper
10	3.49	6.90	− 10.03	17.01
20	2.38	5.69	− 8.77	13.52
30	1.62	4.99	− 8.06	11.40
32	1.55	4.91	− .07	11.17
45	0.87	4.72	− 8.38	10.13
60	0.38	4.63	− 8.68	9.45
64	0.21	4.56	− 8.73	9.15
90	− 0.45	4.47	− 9.20	8.31
120	− 1.60	4.20	− 9.83	6.64
Best window sizes	0.25	3.11	− 5.84	6.35

The negative bias value indicates the average reference respiratory rate was higher than the average estimated respiratory rate

A1. These results endorse that the developed algorithm can estimate respiratory rate close to the reference respiratory rate and thus can be implemented in the commercially available devices that collect PPG signals for long-term continuous respiratory rate measurements.
