# Supplementary figures and images for: Photoplethysmography-Based Respiratory Rate Estimation Algorithm for Health Monitoring Applications
Source: J Med Biol Eng. 2022 Apr 7;42(2):242–52. doi: 10.1007/s40846-022-00700-z (PMC9056464; doi:10.1007/s40846-022-00700-z)

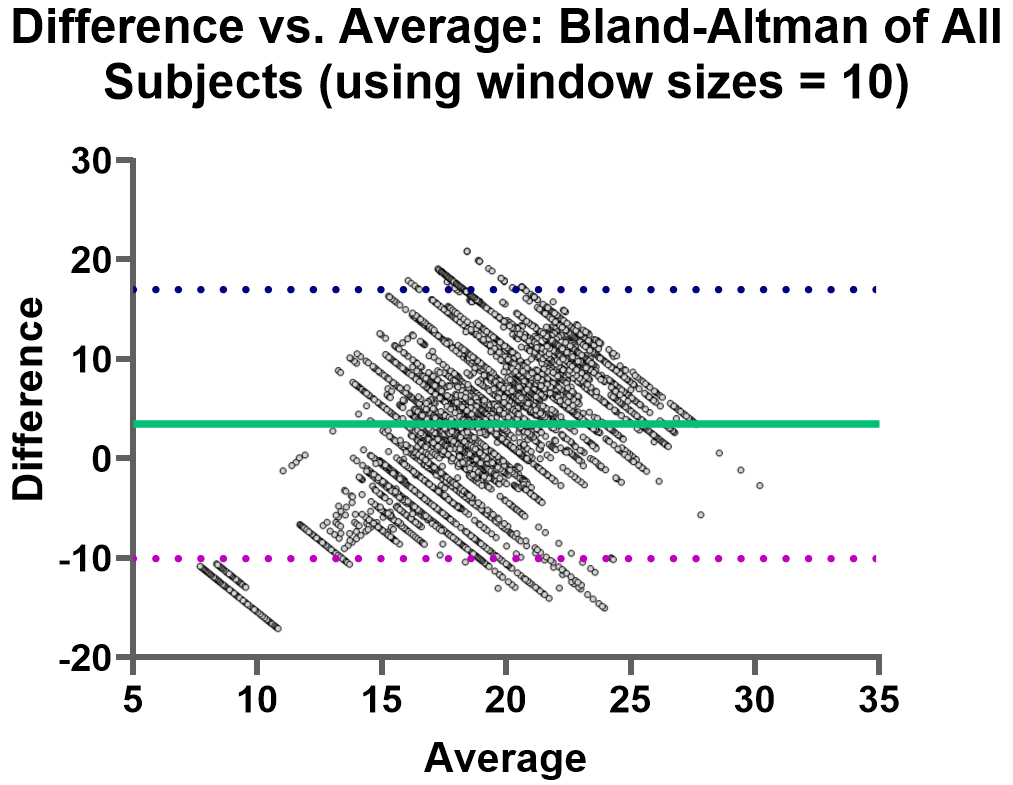

Supplement: Supplementary file 1 — Supplementary file1 (PNG 152 kb) [file 40846_2022_700_MOESM1_ESM.png]

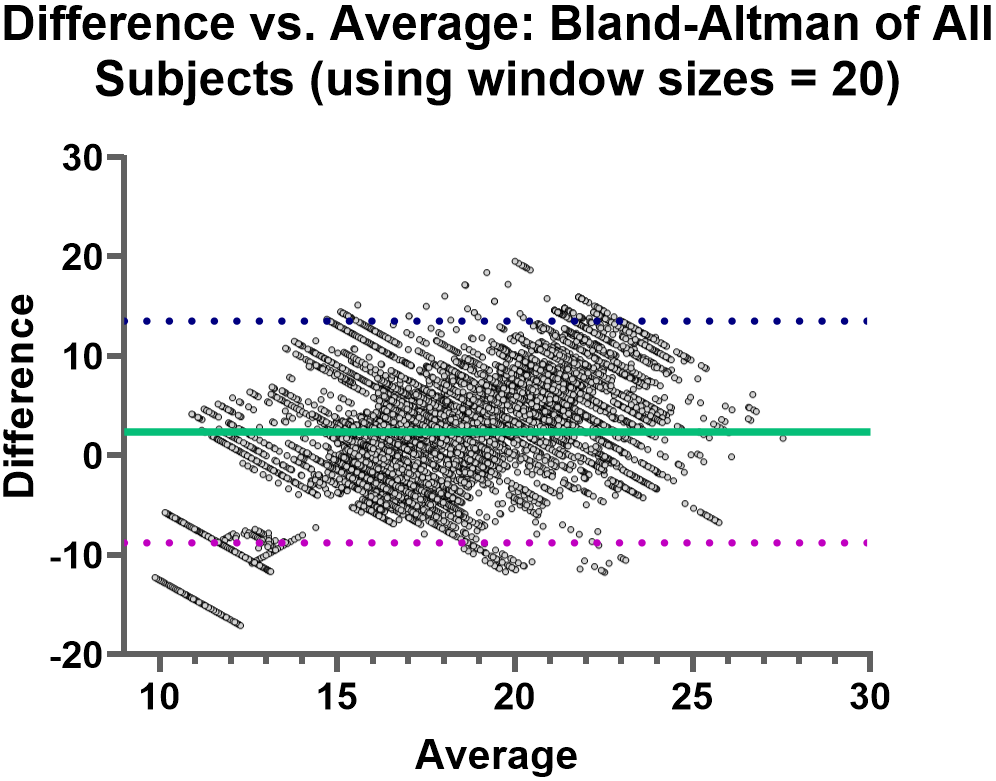

Supplement: Supplementary file 2 — Supplementary file2 (PNG 153 kb) [file 40846_2022_700_MOESM2_ESM.png]

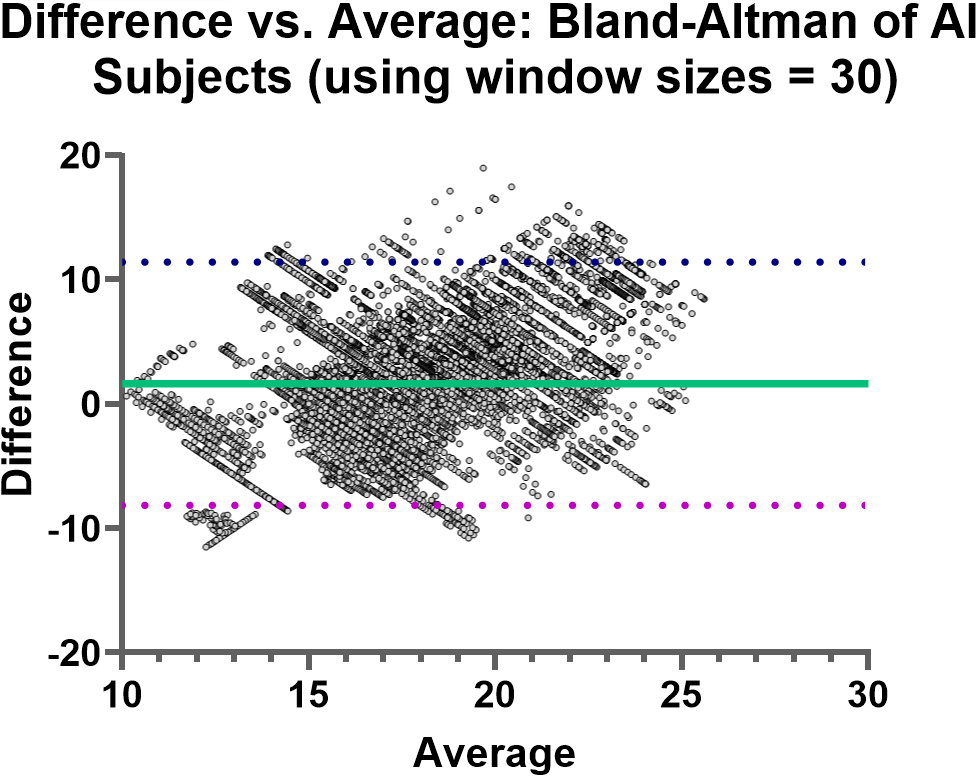

Supplement: Supplementary file 3 — Supplementary file3 (PNG 154 kb) [file 40846_2022_700_MOESM3_ESM.png]

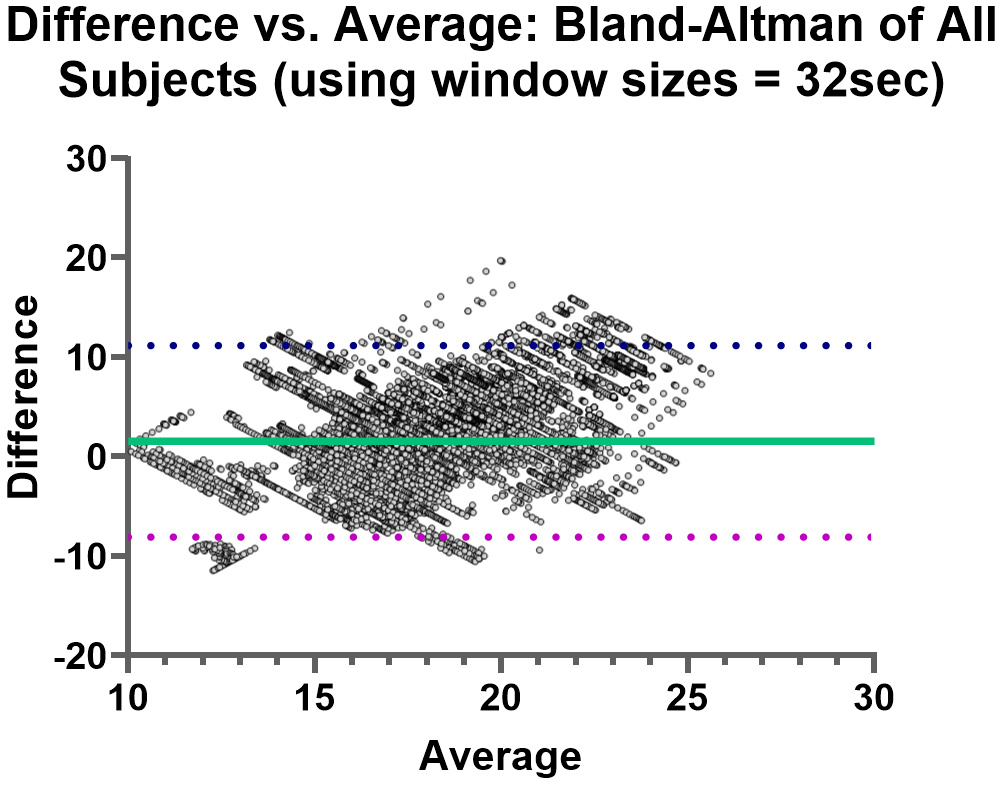

Supplement: Supplementary file 4 — Supplementary file4 (PNG 147 kb) [file 40846_2022_700_MOESM4_ESM.png]

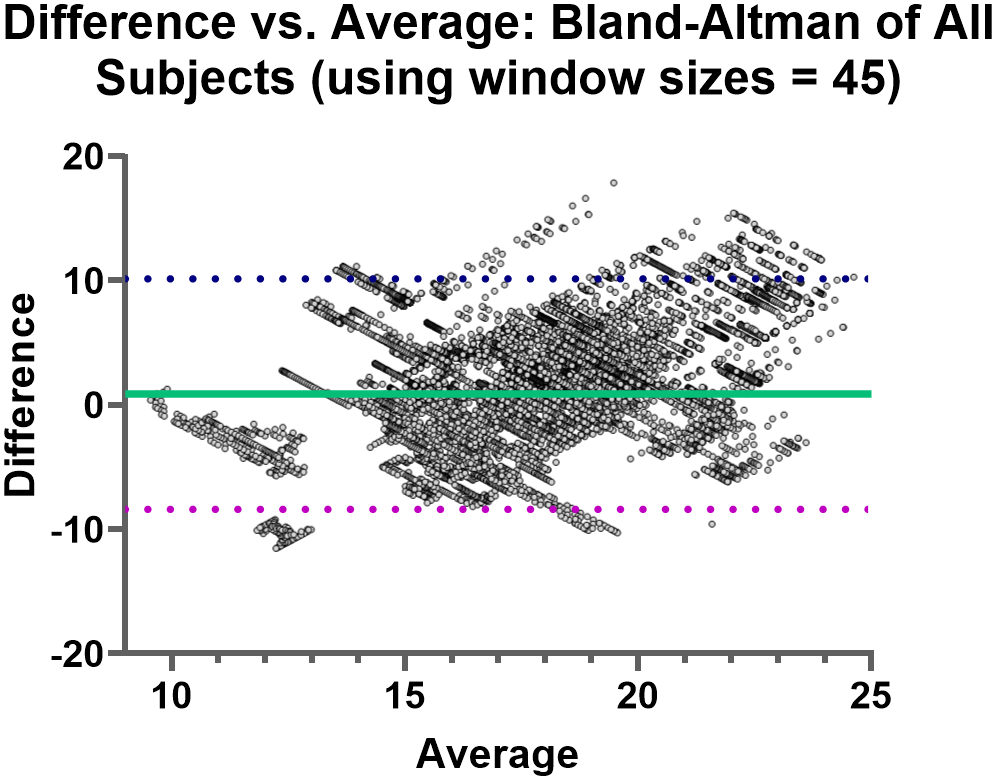

Supplement: Supplementary file 5 — Supplementary file5 (PNG 174 kb) [file 40846_2022_700_MOESM5_ESM.png]

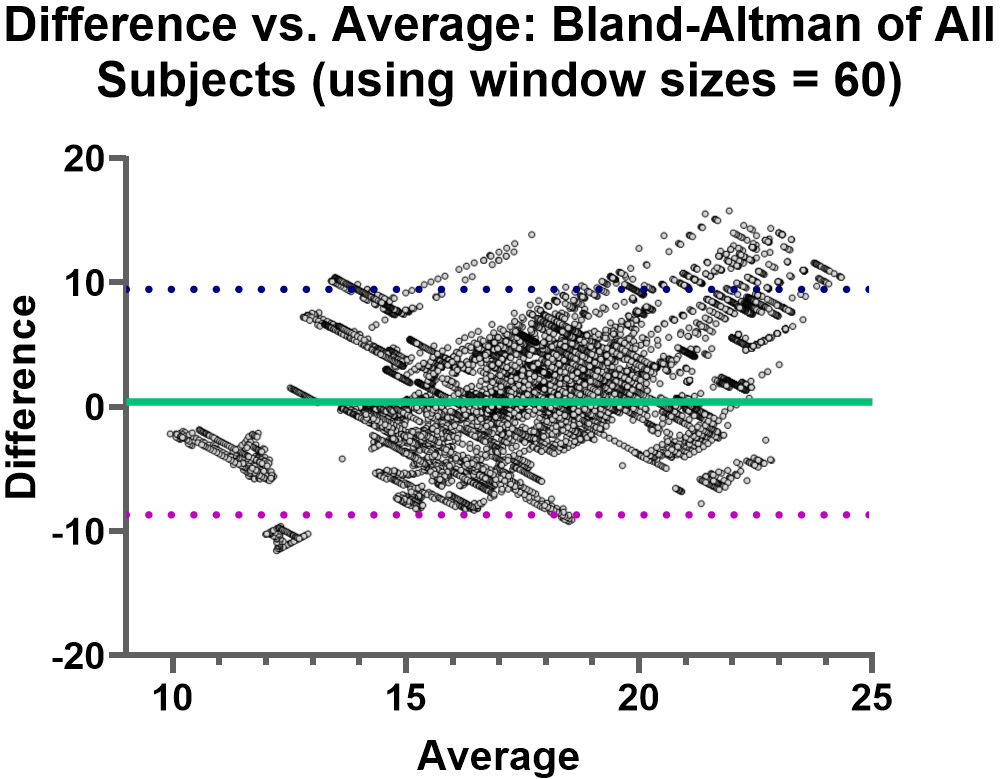

Supplement: Supplementary file 6 — Supplementary file6 (PNG 152 kb) [file 40846_2022_700_MOESM6_ESM.png]

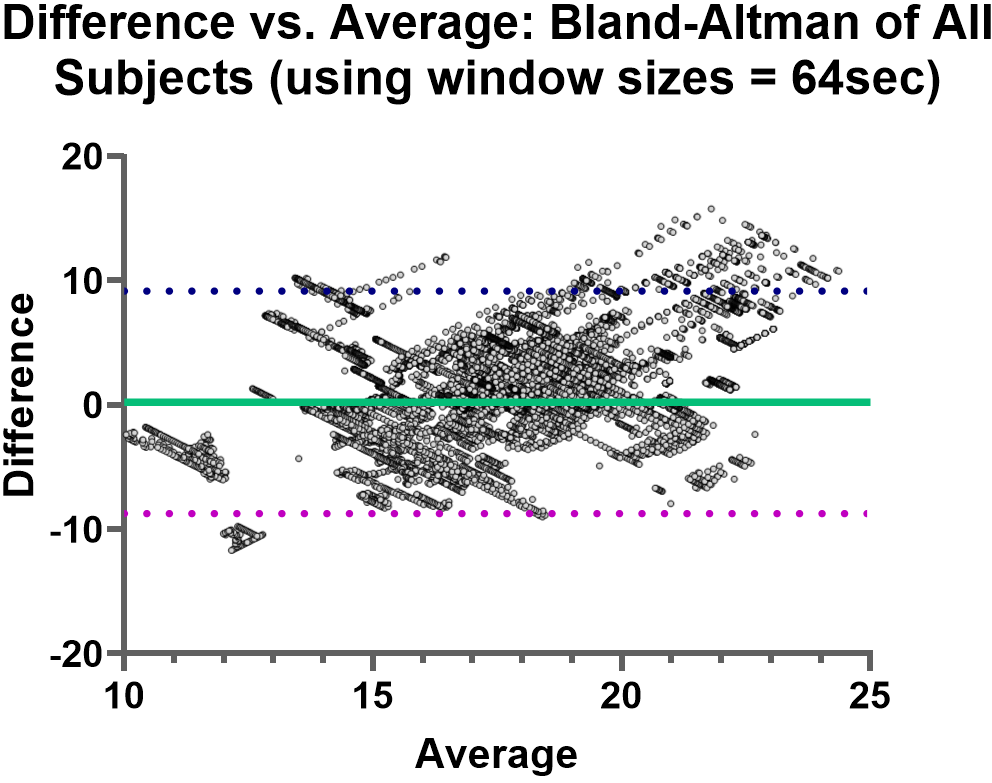

Supplement: Supplementary file 7 — Supplementary file7 (PNG 152 kb) [file 40846_2022_700_MOESM7_ESM.png]

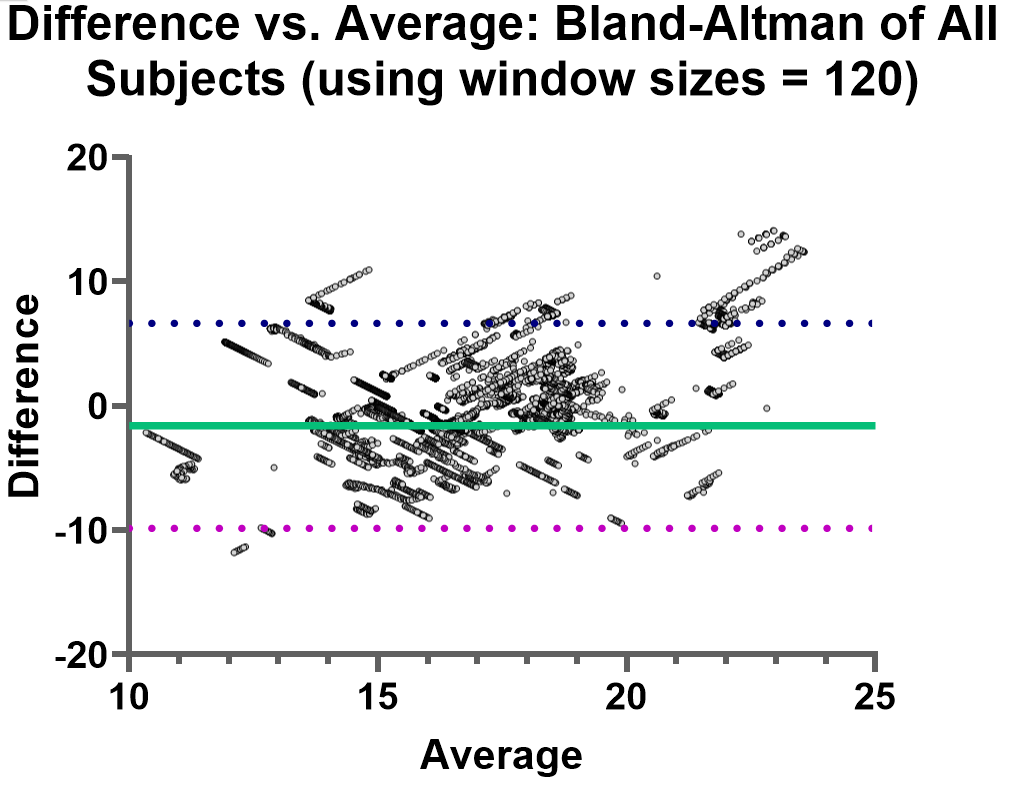

Supplement: Supplementary file 8 — Supplementary file8 (PNG 100 kb) [file 40846_2022_700_MOESM8_ESM.png]

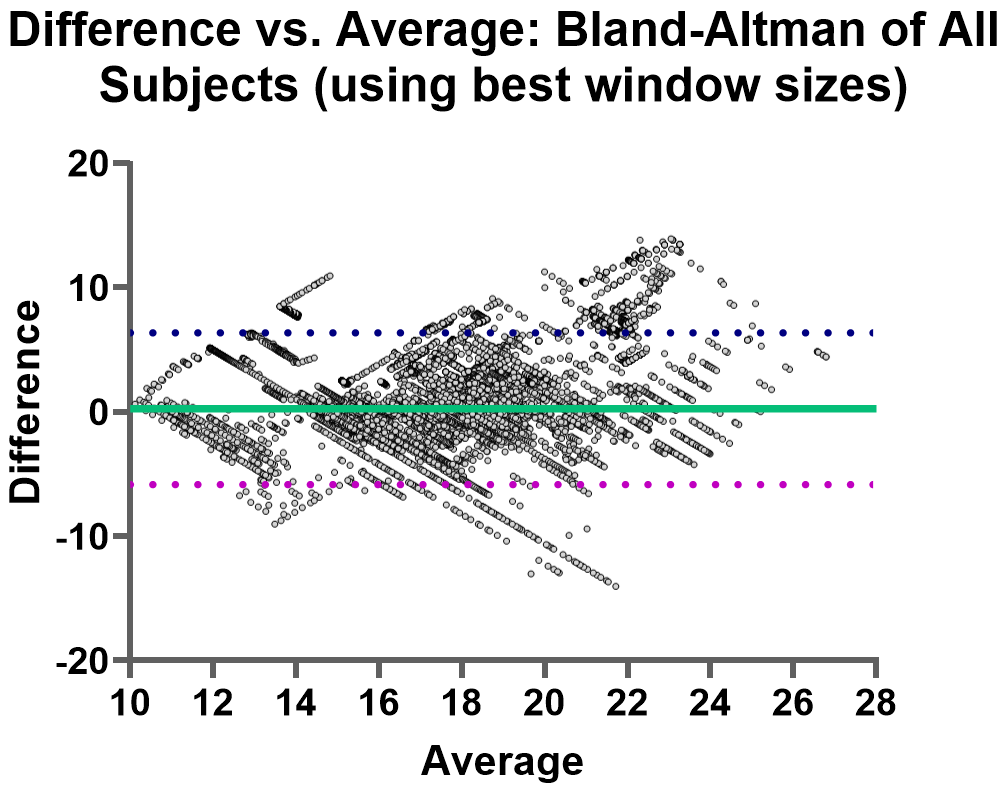

Supplement: Supplementary file 9 — Supplementary file9 (PNG 136 kb) [file 40846_2022_700_MOESM9_ESM.png]
