# Supplementary material for: Photoplethysmography-Based Respiratory Rate Estimation Algorithm for Health Monitoring Applications
Source: J Med Biol Eng. 2022 Apr 7;42(2):242–52. doi: 10.1007/s40846-022-00700-z (PMC9056464; doi:10.1007/s40846-022-00700-z)
Supplement: Supplementary file 10 — Supplementary file10 (PDF 340 kb) [file 40846_2022_700_MOESM10_ESM.pdf]

Table S1. Error in Respiratory Rate Estimation using Different Window Sizes for each Subject

S1	**WIN_10**	**WIN_20**	**WIN_30**	**WIN_45**	**WIN_60**	**WIN_90**	**WIN_120**	**WIN_32**	**WIN_64**
**MAE**	7.23	6.39	1.78	0.84	**0.58**	1.11	2.37	1.62	0.64
**RMSE**	7.31	6.62	2.24	1.14	**0.79**	1.68	3.08	2.15	0.93
S2	**WIN_10**	**WIN_20**	**WIN_30**	**WIN_45**	**WIN_60**	**WIN_90**	**WIN_120**	**WIN_32**	**WIN_64**
**MAE**	7.95	3.30	1.76	0.88	0.50	0.68	1.34	1.56	**0.43**
**RMSE**	8.12	3.45	1.92	1.00	0.63	0.91	1.66	1.69	**0.57**
S3	**WIN_10**	**WIN_20**	**WIN_30**	**WIN_45**	**WIN_60**	**WIN_90**	**WIN_120**	**WIN_32**	**WIN_64**
**MAE**	7.41	2.16	0.61	1.15	1.73	2.64	3.46	**0.60**	1.90
**RMSE**	7.49	2.52	0.88	1.27	1.83	2.69	3.56	**0.79**	1.99
S4	**WIN_10**	**WIN_20**	**WIN_30**	**WIN_45**	**WIN_60**	**WIN_90**	**WIN_120**	**WIN_32**	**WIN_64**
**MAE**	7.11	5.28	4.14	3.07	2.45	**1.83**	2.48	3.85	2.25
**RMSE**	7.83	5.85	5.00	3.76	3.27	**2.61**	2.64	4.64	3.05
S5	**WIN_10**	**WIN_20**	**WIN_30**	**WIN_45**	**WIN_60**	**WIN_90**	**WIN_120**	**WIN_32**	**WIN_64**
**MAE**	12.14	8.26	7.12	6.39	5.44	4.42	**3.40**	7.00	5.19
**RMSE**	12.38	8.63	7.50	6.85	5.73	4.72	**4.00**	7.38	5.42
S6	**WIN_10**	**WIN_20**	**WIN_30**	**WIN_45**	**WIN_60**	**WIN_90**	**WIN_120**	**WIN_32**	**WIN_64**
**MAE**	3.94	**1.39**	3.06	4.20	4.78	5.46	6.04	3.29	4.91
**RMSE**	4.00	**1.46**	3.09	4.22	4.80	5.47	6.06	3.31	4.93
S7	**WIN_10**	**WIN_20**	**WIN_30**	**WIN_45**	**WIN_60**	**WIN_90**	**WIN_120**	**WIN_32**	**WIN_64**
**MAE**		8.15	6.50	5.32	4.58	3.29	**3.06**	6.26	4.36
**RMSE**		8.17	6.53	5.37	4.72	3.83	**3.28**	6.29	4.54
S8	**WIN_10**	**WIN_20**	**WIN_30**	**WIN_45**	**WIN_60**	**WIN_90**	**WIN_120**	**WIN_32**	**WIN_64**
**MAE**		4.66	3.20	2.12	1.54	1.59	2.56	2.96	**1.48**
**RMSE**		4.72	3.27	2.21	1.63	1.66	2.98	3.03	**1.54**
S9	**WIN_10**	**WIN_20**	**WIN_30**	**WIN_45**	**WIN_60**	**WIN_90**	**WIN_120**	**WIN_32**	**WIN_64**
**MAE**		8.51	6.78	5.51	4.71	3.35	**3.11**	6.54	4.49
**RMSE**		8.54	6.82	5.57	4.85	3.95	**3.31**	6.58	4.66
S10	**WIN_10**	**WIN_20**	**WIN_30**	**WIN_45**	**WIN_60**	**WIN_90**	**WIN_120**	**WIN_32**	**WIN_64**
**MAE**	7.51	6.09	4.37	3.12	2.34	**1.61**	1.96	4.08	2.08
**RMSE**	8.56	6.43	4.67	3.33	2.53	**1.74**	2.01	4.33	2.27
S11	**WIN_10**	**WIN_20**	**WIN_30**	**WIN_45**	**WIN_60**	**WIN_90**	**WIN_120**	**WIN_32**	**WIN_64**
**MAE**	7.06	2.80	1.52	**1.00**	1.17	1.90	2.40	1.35	1.30

RMSE	7.41	3.08	2.00	1.68	1.86	2.45	2.83	1.87	1.97
S12	WIN_10	WIN_20	WIN_30	WIN_45	WIN_60	WIN_90	WIN_120	WIN_32	WIN_64
MAE	1.75	3.54	4.89	5.96	6.46	7.18	7.65	5.11	6.56
RMSE	2.31	3.86	5.17	6.14	6.61	7.26	7.73	5.37	6.70
S13	WIN_10	WIN_20	WIN_30	WIN_45	WIN_60	WIN_90	WIN_120	WIN_32	WIN_64
MAE									
RMSE									

S14	WIN_10	WIN_20	WIN_30	WIN_45	WIN_60	WIN_90	WIN_120	WIN_32	WIN_64
MAE	5.40	4.58	3.24	2.29	1.70	2.24	3.12	3.07	1.73
RMSE	6.44	5.74	4.30	3.10	2.31	2.56	3.83	4.11	2.24
S15	WIN_10	WIN_20	WIN_30	WIN_45	WIN_60	WIN_90	WIN_120	WIN_32	WIN_64
MAE	5.40	1.70	1.17	1.36	1.54	1.68	1.83	1.15	1.56
RMSE	5.76	2.23	1.61	1.61	1.81	1.83	1.97	1.53	1.81
S16	WIN_10	WIN_20	WIN_30	WIN_45	WIN_60	WIN_90	WIN_120	WIN_32	WIN_64
MAE	1.14	3.11	4.42	5.30	5.76	6.31	6.69	4.60	5.87
RMSE	1.62	3.21	4.45	5.32	5.77	6.32	6.71	4.62	5.88
S17	WIN_10	WIN_20	WIN_30	WIN_45	WIN_60	WIN_90	WIN_120	WIN_32	WIN_64
MAE	9.01	7.82	6.35	4.76	4.07	2.94	1.88	6.04	3.90
RMSE	9.14	8.17	6.65	4.95	4.26	3.23	2.47	6.31	4.10
S18	WIN_10	WIN_20	WIN_30	WIN_45	WIN_60	WIN_90	WIN_120	WIN_32	WIN_64
MAE	6.97	9.39	10.19	10.72	10.91	10.98	10.79	10.29	10.93
RMSE	7.04	9.41	10.20	10.73	10.92	11.00	10.83	10.30	10.94
S19	WIN_10	WIN_20	WIN_30	WIN_45	WIN_60	WIN_90	WIN_120	WIN_32	WIN_64
MAE	6.96	2.15	2.12	3.00	3.78	4.35	4.43	2.29	3.87
RMSE	7.80	2.77	2.62	3.35	4.01	4.54	4.60	2.74	4.08
S20	WIN_10	WIN_20	WIN_30	WIN_45	WIN_60	WIN_90	WIN_120	WIN_32	WIN_64
MAE	11.39	7.46	5.78	4.60	3.89	2.98	1.89	5.55	3.74
RMSE	11.50	7.68	5.93	4.70	3.96	3.15	2.39	5.69	3.80
S21	WIN_10	WIN_20	WIN_30	WIN_45	WIN_60	WIN_90	WIN_120	WIN_32	WIN_64
MAE	2.18	2.37	3.11	3.84	4.26	4.90	5.56	3.22	4.37
RMSE	2.96	2.75	3.49	4.10	4.46	5.05	5.68	3.59	4.56
S22	WIN_10	WIN_20	WIN_30	WIN_45	WIN_60	WIN_90	WIN_120	WIN_32	WIN_64

**MAE**	**2.37**	4.17	5.81	6.90	7.48	8.04	8.32	6.03	7.59
**RMSE**	**3.05**	4.22	5.84	6.92	7.49	8.05	8.33	6.05	7.60
S23	**WIN_10**	**WIN_20**	**WIN_30**	**WIN_45**	**WIN_60**	**WIN_90**	**WIN_120**	**WIN_32**	**WIN_64**
**MAE**		14.07	7.88	6.73	7.06	6.98	**4.24**	6.97	6.83
**RMSE**		14.11	8.34	7.84	8.02	7.36	**5.08**	8.08	7.73
S24	**WIN_10**	**WIN_20**	**WIN_30**	**WIN_45**	**WIN_60**	**WIN_90**	**WIN_120**	**WIN_32**	**WIN_64**
**MAE**	3.80	**1.14**	1.40	2.20	2.81	3.82	5.15	1.44	3.00
**RMSE**	4.33	**1.54**	1.67	2.34	2.91	3.95	5.44	1.68	3.09
S25	**WIN_10**	**WIN_20**	**WIN_30**	**WIN_45**	**WIN_60**	**WIN_90**	**WIN_120**	**WIN_32**	**WIN_64**
**MAE**	3.23	**0.85**	1.64	2.51	3.00	3.43	3.84	1.80	3.09
**RMSE**	3.85	**1.14**	1.88	2.65	3.09	3.51	3.93	2.01	3.18
S26	**WIN_10**	**WIN_20**	**WIN_30**	**WIN_45**	**WIN_60**	**WIN_90**	**WIN_120**	**WIN_32**	**WIN_64**
**MAE**	13.84	9.62	7.51	5.59	5.52	**4.31**	4.59	6.63	5.10
**RMSE**	13.99	9.88	7.98	7.26	7.10	**5.77**	5.59	7.66	6.75

S27	**WIN_10**	**WIN_20**	**WIN_30**	**WIN_45**	**WIN_60**	**WIN_90**	**WIN_120**	**WIN_32**	**WIN_64**
**MAE**	16.34	11.48	9.96	9.09	8.43	7.59	**6.58**	9.88	8.21
**RMSE**	16.41	11.56	10.05	9.22	8.55	7.78	**6.86**	10.00	8.33
S28	**WIN_10**	**WIN_20**	**WIN_30**	**WIN_45**	**WIN_60**	**WIN_90**	**WIN_120**	**WIN_32**	**WIN_64**
**MAE**	6.66	4.99	3.62	2.07	1.39	**1.25**	1.92	3.27	1.23
**RMSE**	6.89	5.57	4.12	2.34	1.60	**1.35**	2.10	3.76	1.46
S29	**WIN_10**	**WIN_20**	**WIN_30**	**WIN_45**	**WIN_60**	**WIN_90**	**WIN_120**	**WIN_32**	**WIN_64**
**MAE**	6.21	4.37	2.83	1.93	1.29	**1.12**	2.02	2.92	1.15
**RMSE**	6.98	4.90	3.28	2.48	1.74	**1.45**	2.22	3.53	1.61
S30	**WIN_10**	**WIN_20**	**WIN_30**	**WIN_45**	**WIN_60**	**WIN_90**	**WIN_120**	**WIN_32**	**WIN_64**
**MAE**	7.42	3.98	2.34	1.43	1.05	1.00	1.86	2.14	**0.95**
**RMSE**	7.76	4.31	2.72	1.71	1.35	1.37	2.53	2.52	**1.24**
S31	**WIN_10**	**WIN_20**	**WIN_30**	**WIN_45**	**WIN_60**	**WIN_90**	**WIN_120**	**WIN_32**	**WIN_64**
**MAE**	8.71	3.69	1.96	0.92	0.54	0.73	1.72	1.72	**0.51**
**RMSE**	8.81	3.86	2.17	1.11	0.67	0.96	2.04	1.93	**0.60**
S32	**WIN_10**	**WIN_20**	**WIN_30**	**WIN_45**	**WIN_60**	**WIN_90**	**WIN_120**	**WIN_32**	**WIN_64**
**MAE**	2.62	**2.53**	3.94	5.16	5.91	6.80	7.78	4.17	6.09
**RMSE**	2.89	**2.68**	4.10	5.23	5.96	6.87	7.92	4.32	6.13

S33	WIN_10	WIN_20	WIN_30	WIN_45	WIN_60	WIN_90	WIN_120	WIN_32	WIN_64
**MAE**									
**RMSE**									
S34	**WIN_10**	**WIN_20**	**WIN_30**	**WIN_45**	**WIN_60**	**WIN_90**	**WIN_120**	**WIN_32**	**WIN_64**
**MAE**	10.10	5.93	4.13	2.93	2.32	**1.46**	1.59	3.84	2.16
**RMSE**	10.39	6.17	4.39	3.18	2.56	**1.84**	1.68	4.07	2.40
S35	**WIN_10**	**WIN_20**	**WIN_30**	**WIN_45**	**WIN_60**	**WIN_90**	**WIN_120**	**WIN_32**	**WIN_64**
**MAE**	6.52	3.01	1.91	1.11	**0.96**	1.20	1.63	1.73	0.98
**RMSE**	6.90	3.66	2.83	1.75	**1.31**	1.39	1.98	2.64	1.27
S36	**WIN_10**	**WIN_20**	**WIN_30**	**WIN_45**	**WIN_60**	**WIN_90**	**WIN_120**	**WIN_32**	**WIN_64**
**MAE**	7.98	4.79	2.76	1.38	**0.70**	0.72	1.49	2.48	0.60
**RMSE**	8.09	4.89	2.88	1.56	**0.95**	0.89	1.87	2.61	0.83
S37	**WIN_10**	**WIN_20**	**WIN_30**	**WIN_45**	**WIN_60**	**WIN_90**	**WIN_120**	**WIN_32**	**WIN_64**
**MAE**	7.36	2.91	1.26	**0.72**	0.90	1.54	2.55	1.08	1.01
**RMSE**	7.52	3.32	1.66	**0.86**	1.10	1.94	3.01	1.46	1.23
S38	**WIN_10**	**WIN_20**	**WIN_30**	**WIN_45**	**WIN_60**	**WIN_90**	**WIN_120**	**WIN_32**	**WIN_64**
**MAE**	7.74	6.40	5.20	3.57	2.67	1.57	**1.06**	4.85	2.41
**RMSE**	9.00	6.99	6.03	4.16	3.16	1.84	**1.24**	5.70	2.83
S39	**WIN_10**	**WIN_20**	**WIN_30**	**WIN_45**	**WIN_60**	**WIN_90**	**WIN_120**	**WIN_32**	**WIN_64**
**MAE**	10.24	8.77	7.03	5.74	5.05	4.13	**2.94**	6.77	4.91
**RMSE**	11.12	9.10	7.36	5.93	5.16	4.33	**3.38**	7.07	5.04

S40	WIN_10	WIN_20	WIN_30	WIN_45	WIN_60	WIN_90	WIN_120	WIN_32	WIN_64
**MAE**	3.53	3.60	3.80	2.34	1.72	1.70	2.77	3.72	**1.58**
**RMSE**	4.46	4.83	5.11	3.05	2.30	2.06	2.97	4.97	**2.12**
S41	**WIN_10**	**WIN_20**	**WIN_30**	**WIN_45**	**WIN_60**	**WIN_90**	**WIN_120**	**WIN_32**	**WIN_64**
**MAE**	9.30	6.02	4.55	4.57	4.68	5.27	**4.30**	4.32	4.67
**RMSE**	9.88	6.48	5.24	5.42	5.62	6.10	**4.87**	5.02	5.64
S42	**WIN_10**	**WIN_20**	**WIN_30**	**WIN_45**	**WIN_60**	**WIN_90**	**WIN_120**	**WIN_32**	**WIN_64**
**MAE**	1.59	1.09	0.90	0.75	**0.73**	0.96	1.43	0.86	0.75
**RMSE**	1.81	1.22	1.01	0.84	**0.79**	1.00	1.58	0.94	0.80
S43	**WIN_10**	**WIN_20**	**WIN_30**	**WIN_45**	**WIN_60**	**WIN_90**	**WIN_120**	**WIN_32**	**WIN_64**
**MAE**	7.59	3.45	1.99	1.20	**1.02**	1.45	2.27	1.77	1.02
**RMSE**	7.92	4.03	2.46	1.44	**1.34**	1.88	2.65	2.21	1.38

S44	**WIN_10**	**WIN_20**	**WIN_30**	**WIN_45**	**WIN_60**	**WIN_90**	**WIN_120**	**WIN_32**	**WIN_64**
**MAE**	**1.35**	1.47	1.64	1.74	1.68	1.83	1.49	1.60	1.64
**RMSE**	**2.05**	2.58	2.99	3.19	2.84	2.79	1.91	2.91	2.74
S45	**WIN_10**	**WIN_20**	**WIN_30**	**WIN_45**	**WIN_60**	**WIN_90**	**WIN_120**	**WIN_32**	**WIN_64**
**MAE**	6.15	8.79	9.07	8.33	8.31	**5.27**	5.78	8.49	7.23
**RMSE**	7.55	9.62	9.76	9.34	9.26	**7.75**	6.57	9.35	8.61
S46	**WIN_10**	**WIN_20**	**WIN_30**	**WIN_45**	**WIN_60**	**WIN_90**	**WIN_120**	**WIN_32**	**WIN_64**
**MAE**	**3.99**	4.24	4.32	4.24	4.06	4.16	4.56	4.33	4.05
**RMSE**	**5.22**	5.29	5.27	5.05	4.63	4.49	4.69	5.26	4.57
S47	**WIN_10**	**WIN_20**	**WIN_30**	**WIN_45**	**WIN_60**	**WIN_90**	**WIN_120**	**WIN_32**	**WIN_64**
**MAE**	**2.62**	3.16	3.40	3.57	3.84	4.37	5.30	3.46	3.94
**RMSE**	**2.94**	3.28	3.46	3.61	3.87	4.48	5.53	3.50	3.97
S48	**WIN_10**	**WIN_20**	**WIN_30**	**WIN_45**	**WIN_60**	**WIN_90**	**WIN_120**	**WIN_32**	**WIN_64**
**MAE**	2.69	2.66	**2.70**	2.71	2.80	3.15	3.72	2.71	2.88
**RMSE**	3.14	3.04	**3.03**	3.00	3.08	3.51	4.12	3.04	3.18
S49	**WIN_10**	**WIN_20**	**WIN_30**	**WIN_45**	**WIN_60**	**WIN_90**	**WIN_120**	**WIN_32**	**WIN_64**
**MAE**	8.33	6.80	4.98	3.57	2.81	**1.47**	1.66	4.73	2.51
**RMSE**	9.34	7.06	5.28	3.88	3.11	**1.87**	1.72	5.02	2.85
S50	**WIN_10**	**WIN_20**	**WIN_30**	**WIN_45**	**WIN_60**	**WIN_90**	**WIN_120**	**WIN_32**	**WIN_64**
**MAE**	12.08	10.33	8.67	8.11	7.50	6.49	**4.79**	8.98	7.34
**RMSE**	12.12	10.45	8.83	8.34	7.69	6.77	**5.38**	9.24	7.54
S51	**WIN_10**	**WIN_20**	**WIN_30**	**WIN_45**	**WIN_60**	**WIN_90**	**WIN_120**	**WIN_32**	**WIN_64**
**MAE**		7.51	9.64	8.35	7.54	6.11	**3.88**	9.39	7.30
**RMSE**		8.20	9.67	8.40	7.67	6.59	**5.26**	9.42	7.47
S52	**WIN_10**	**WIN_20**	**WIN_30**	**WIN_45**	**WIN_60**	**WIN_90**	**WIN_120**	**WIN_32**	**WIN_64**
**MAE**	**4.91**	5.16	5.27	5.37	5.41	5.52	5.72	5.30	5.43
**RMSE**	**4.96**	5.18	5.29	5.37	5.41	5.53	5.74	5.31	5.44

S53	**WIN_10**	**WIN_20**	**WIN_30**	**WIN_45**	**WIN_60**	**WIN_90**	**WIN_120**	**WIN_32**	**WIN_64**
**MAE**	2.88	2.10	1.77	1.83	1.96	2.02	2.48	**1.77**	2.03
**RMSE**	3.89	2.94	2.25	2.13	2.21	2.24	2.82	**2.19**	2.26
