# Supplementary material for: Photoplethysmography-Based Respiratory Rate Estimation Algorithm for Health Monitoring Applications
Source: J Med Biol Eng. 2022 Apr 7;42(2):242–52. doi: 10.1007/s40846-022-00700-z (PMC9056464; doi:10.1007/s40846-022-00700-z)
Supplement: Supplementary file 11 — Supplementary file11 (PDF 339 kb) [file 40846_2022_700_MOESM11_ESM.pdf]

Table S2. Error in Respiratory Rate Estimation using Different Window Sizes for each subject (removing points with SQI = nan)

S1	**WIN_10**	**WIN_20**	**WIN_30**	**WIN_45**	**WIN_60**	**WIN_90**	**WIN_120**	**WIN_32**	**WIN_64**
**MAE**	7.23	6.37	1.74	0.84	**0.61**	1.18	2.46	1.62	0.64
**RMSE**	7.31	6.60	2.22	1.13	**0.83**	1.77	3.16	2.15	0.93
S2	**WIN_10**	**WIN_20**	**WIN_30**	**WIN_45**	**WIN_60**	**WIN_90**	**WIN_120**	**WIN_32**	**WIN_64**
**MAE**	7.95	3.30	1.76	0.88	0.50	0.68	1.34	1.56	**0.43**
**RMSE**	8.12	3.45	1.92	1.00	0.63	0.91	1.66	1.69	**0.57**
S3	**WIN_10**	**WIN_20**	**WIN_30**	**WIN_45**	**WIN_60**	**WIN_90**	**WIN_120**	**WIN_32**	**WIN_64**
**MAE**	7.41	2.16	0.61	1.15	1.73	2.64	3.46	**0.60**	1.90
**RMSE**	7.49	2.52	0.88	1.27	1.83	2.69	3.56	**0.79**	1.99
S4	**WIN_10**	**WIN_20**	**WIN_30**	**WIN_45**	**WIN_60**	**WIN_90**	**WIN_120**	**WIN_32**	**WIN_64**
**MAE**	7.11	5.28	4.14	3.07	2.45	**1.83**	2.48	3.85	2.25
**RMSE**	7.83	5.85	5.00	3.76	3.27	**2.61**	2.64	4.64	3.05
S5	**WIN_10**	**WIN_20**	**WIN_30**	**WIN_45**	**WIN_60**	**WIN_90**	**WIN_120**	**WIN_32**	**WIN_64**
**MAE**	12.14	8.01	6.64	5.78	5.11	4.42	**3.40**	6.55	5.01
**RMSE**	12.38	8.26	6.83	6.04	5.31	4.72	**4.00**	6.76	5.21
S6	**WIN_10**	**WIN_20**	**WIN_30**	**WIN_45**	**WIN_60**	**WIN_90**	**WIN_120**	**WIN_32**	**WIN_64**
**MAE**	3.94	**1.39**	3.06	4.20	4.78	5.46	6.04	3.29	4.91
**RMSE**	4.00	**1.46**	3.09	4.22	4.80	5.47	6.06	3.31	4.93
S7	**WIN_10**	**WIN_20**	**WIN_30**	**WIN_45**	**WIN_60**	**WIN_90**	**WIN_120**	**WIN_32**	**WIN_64**
**MAE**		8.15	6.50	5.32	4.58	3.29	**3.06**	6.26	4.36
**RMSE**		8.17	6.53	5.37	4.72	3.83	**3.28**	6.29	4.54
S8	**WIN_10**	**WIN_20**	**WIN_30**	**WIN_45**	**WIN_60**	**WIN_90**	**WIN_120**	**WIN_32**	**WIN_64**
**MAE**		4.66	3.20	2.12	1.54	1.59	2.56	2.96	**1.48**
**RMSE**		4.72	3.27	2.21	1.63	1.66	2.98	3.03	**1.54**
S9	**WIN_10**	**WIN_20**	**WIN_30**	**WIN_45**	**WIN_60**	**WIN_90**	**WIN_120**	**WIN_32**	**WIN_64**
**MAE**		8.51	6.78	5.51	4.71	3.35	**3.11**	6.54	4.49
**RMSE**		8.54	6.82	5.57	4.85	3.95	**3.31**	6.58	4.66
S10	**WIN_10**	**WIN_20**	**WIN_30**	**WIN_45**	**WIN_60**	**WIN_90**	**WIN_120**	**WIN_32**	**WIN_64**
**MAE**	7.88	5.92	4.15	3.04	2.41	1.81	**1.55**	3.95	2.27
**RMSE**	8.81	6.22	4.39	3.23	2.60	1.90	**1.56**	4.17	2.44
S11	**WIN_10**	**WIN_20**	**WIN_30**	**WIN_45**	**WIN_60**	**WIN_90**	**WIN_120**	**WIN_32**	**WIN_64**
**MAE**	7.06	2.80	1.52	**1.00**	1.17	1.90	2.40	1.35	1.30

RMSE	7.41	3.08	2.00	1.68	1.86	2.45	2.83	1.87	1.97
S12	WIN_10	WIN_20	WIN_30	WIN_45	WIN_60	WIN_90	WIN_120	WIN_32	WIN_64
MAE	1.75	3.54	4.89	5.96	6.46	7.18	7.65	5.11	6.56
RMSE	2.31	3.86	5.17	6.14	6.61	7.26	7.73	5.37	6.70
S13	WIN_10	WIN_20	WIN_30	WIN_45	WIN_60	WIN_90	WIN_120	WIN_32	WIN_64
MAE									
RMSE									
S14	WIN_10	WIN_20	WIN_30	WIN_45	WIN_60	WIN_90	WIN_120	WIN_32	WIN_64
MAE	5.58	2.33	2.54					2.83	
RMSE	6.42	3.00	3.30					3.56	
S15	WIN_10	WIN_20	WIN_30	WIN_45	WIN_60	WIN_90	WIN_120	WIN_32	WIN_64
MAE	5.44	1.70	1.15	1.33	1.52	1.71	1.91	1.15	1.56
RMSE	5.82	2.21	1.59	1.59	1.79	1.86	2.06	1.53	1.82
S16	WIN_10	WIN_20	WIN_30	WIN_45	WIN_60	WIN_90	WIN_120	WIN_32	WIN_64
MAE	1.14	3.16	4.44	5.31	5.76	6.31	6.69	4.62	5.87
RMSE	1.62	3.25	4.47	5.33	5.77	6.32	6.71	4.64	5.88
S17	WIN_10	WIN_20	WIN_30	WIN_45	WIN_60	WIN_90	WIN_120	WIN_32	WIN_64
MAE	9.03	7.82	6.42	4.83	4.07	2.94	1.88	6.12	3.90
RMSE	9.17	8.18	6.73	5.01	4.26	3.23	2.47	6.39	4.10
S18	WIN_10	WIN_20	WIN_30	WIN_45	WIN_60	WIN_90	WIN_120	WIN_32	WIN_64
MAE	6.97	9.39	10.19	10.72	10.91	10.98	10.79	10.29	10.93
RMSE	7.04	9.41	10.19	10.73	10.92	11.00	10.83	10.30	10.94
S19	WIN_10	WIN_20	WIN_30	WIN_45	WIN_60	WIN_90	WIN_120	WIN_32	WIN_64
MAE	6.78	2.14	2.17	3.02	3.74	4.27	4.28	2.29	3.87
RMSE	7.65	2.73	2.65	3.35	3.96	4.46	4.46	2.74	4.08
S20	WIN_10	WIN_20	WIN_30	WIN_45	WIN_60	WIN_90	WIN_120	WIN_32	WIN_64
MAE	11.46	7.34	5.68	4.54	3.89	2.98	1.88	5.45	3.74
RMSE	11.56	7.54	5.83	4.64	3.96	3.15	2.39	5.59	3.80
S21	WIN_10	WIN_20	WIN_30	WIN_45	WIN_60	WIN_90	WIN_120	WIN_32	WIN_64
MAE	2.02	2.34	3.30	4.17	4.77	5.53	5.83	3.44	4.95
RMSE	2.65	2.74	3.63	4.40	4.91	5.60	5.92	3.75	5.06
S22	WIN_10	WIN_20	WIN_30	WIN_45	WIN_60	WIN_90	WIN_120	WIN_32	WIN_64

**MAE**	**2.37**	4.17	5.81	6.90	7.48	8.04	8.32	6.03	7.59
**RMSE**	**3.05**	4.22	5.84	6.92	7.49	8.05	8.33	6.05	7.60
S23	**WIN_10**	**WIN_20**	**WIN_30**	**WIN_45**	**WIN_60**	**WIN_90**	**WIN_120**	**WIN_32**	**WIN_64**
**MAE**		13.87	9.09	8.11	6.63	6.01	**3.99**	8.96	6.28
**RMSE**		13.92	9.43	8.57	7.41	6.25	**4.79**	9.54	7.05
S24	**WIN_10**	**WIN_20**	**WIN_30**	**WIN_45**	**WIN_60**	**WIN_90**	**WIN_120**	**WIN_32**	**WIN_64**
**MAE**	3.80	**1.14**	1.40	2.20	2.81	3.82	5.15	1.44	3.00
**RMSE**	4.33	**1.54**	1.67	2.34	2.91	3.95	5.44	1.68	3.09
S25	**WIN_10**	**WIN_20**	**WIN_30**	**WIN_45**	**WIN_60**	**WIN_90**	**WIN_120**	**WIN_32**	**WIN_64**
**MAE**	3.15	**0.85**	1.64	2.58	3.13	3.70	4.58	1.80	3.23
**RMSE**	3.74	**1.15**	1.89	2.72	3.22	3.76	4.60	2.01	3.32
S26	**WIN_10**	**WIN_20**	**WIN_30**	**WIN_45**	**WIN_60**	**WIN_90**	**WIN_120**	**WIN_32**	**WIN_64**
**MAE**	13.86	9.68	7.99	6.82	6.37	4.91	**2.86**	6.63	5.93
**RMSE**	14.01	9.93	8.43	8.06	7.56	5.85	**3.01**	7.66	7.17
S27	**WIN_10**	**WIN_20**	**WIN_30**	**WIN_45**	**WIN_60**	**WIN_90**	**WIN_120**	**WIN_32**	**WIN_64**
**MAE**	16.39	11.50	9.95	8.99	8.57	8.04	**7.60**	9.88	8.49
**RMSE**	16.45	11.57	10.01	9.06	8.63	8.10	**7.71**	10.00	8.55
S28	**WIN_10**	**WIN_20**	**WIN_30**	**WIN_45**	**WIN_60**	**WIN_90**	**WIN_120**	**WIN_32**	**WIN_64**
**MAE**	6.66	4.99	3.62	2.07	1.39	1.25	1.92	3.27	**1.23**
**RMSE**	6.89	5.57	4.12	2.34	1.60	1.35	2.10	3.76	**1.46**
S29	**WIN_10**	**WIN_20**	**WIN_30**	**WIN_45**	**WIN_60**	**WIN_90**	**WIN_120**	**WIN_32**	**WIN_64**
**MAE**	6.30	4.34	2.60	1.49	0.85	0.77	2.75	2.40	**0.68**
**RMSE**	7.03	4.76	2.92	1.76	0.99	1.06	2.80	2.68	**0.80**
S30	**WIN_10**	**WIN_20**	**WIN_30**	**WIN_45**	**WIN_60**	**WIN_90**	**WIN_120**	**WIN_32**	**WIN_64**
**MAE**	7.47	3.86	2.26	1.42	1.05	1.00	1.87	2.08	**0.95**
**RMSE**	7.81	4.18	2.66	1.71	1.35	1.37	2.53	2.47	**1.24**
S31	**WIN_10**	**WIN_20**	**WIN_30**	**WIN_45**	**WIN_60**	**WIN_90**	**WIN_120**	**WIN_32**	**WIN_64**
**MAE**	8.71	3.69	1.96	0.92	0.54	0.73	1.72	1.72	**0.51**
**RMSE**	8.81	3.86	2.17	1.11	0.67	0.96	2.04	1.93	**0.60**
S32	**WIN_10**	**WIN_20**	**WIN_30**	**WIN_45**	**WIN_60**	**WIN_90**	**WIN_120**	**WIN_32**	**WIN_64**
**MAE**	2.62	**2.53**	3.94	5.16	5.91	6.80	7.78	4.17	6.09
**RMSE**	2.89	**2.68**	4.10	5.23	5.96	6.87	7.92	4.32	6.13

S33	WIN_10	WIN_20	WIN_30	WIN_45	WIN_60	WIN_90	WIN_120	WIN_32	WIN_64
MAE									
RMSE									
S34	WIN_10	WIN_20	WIN_30	WIN_45	WIN_60	WIN_90	WIN_120	WIN_32	WIN_64
MAE	10.21	5.88	4.09	2.77	2.03	1.07	1.21	3.80	1.83
RMSE	10.50	6.09	4.34	3.02	2.25	1.34	1.23	4.01	2.03
S35	WIN_10	WIN_20	WIN_30	WIN_45	WIN_60	WIN_90	WIN_120	WIN_32	WIN_64
MAE	6.59	2.99	1.60	0.79	0.85	1.46	2.73	1.73	0.92
RMSE	6.96	3.50	1.98	0.96	1.01	1.67	2.74	2.64	1.08
S36	WIN_10	WIN_20	WIN_30	WIN_45	WIN_60	WIN_90	WIN_120	WIN_32	WIN_64
MAE	7.98	4.79	2.76	1.38	0.70	0.72	1.49	2.48	0.60
RMSE	8.09	4.89	2.88	1.56	0.95	0.89	1.87	2.61	0.83
S37	WIN_10	WIN_20	WIN_30	WIN_45	WIN_60	WIN_90	WIN_120	WIN_32	WIN_64
MAE	7.38	2.95	1.34	0.73	0.90	1.75	2.95	1.08	1.01
RMSE	7.53	3.33	1.72	0.88	1.13	2.21	3.36	1.46	1.27
S38	WIN_10	WIN_20	WIN_30	WIN_45	WIN_60	WIN_90	WIN_120	WIN_32	WIN_64
MAE	7.66	6.24	5.02	3.40	2.46	1.23	1.15	4.85	2.17
RMSE	8.91	6.83	5.91	4.06	3.01	1.43	1.35	5.70	2.63
S39	WIN_10	WIN_20	WIN_30	WIN_45	WIN_60	WIN_90	WIN_120	WIN_32	WIN_64
MAE	10.24	8.77	7.03	5.74	5.05	4.13	2.94	6.77	4.91
RMSE	11.12	9.10	7.36	5.93	5.16	4.33	3.38	7.07	5.04
S40	WIN_10	WIN_20	WIN_30	WIN_45	WIN_60	WIN_90	WIN_120	WIN_32	WIN_64
MAE	3.03	3.09	3.07	2.24	0.85	0.47	2.89	3.03	0.65
RMSE	3.73	3.69	3.49	2.51	0.88	0.47	2.90	3.41	0.67
S41	WIN_10	WIN_20	WIN_30	WIN_45	WIN_60	WIN_90	WIN_120	WIN_32	WIN_64
MAE	9.37	5.95	4.45	4.44	4.73	4.68	3.17	4.21	4.77
RMSE	9.94	6.42	5.19	5.34	5.69	5.32	3.69	4.96	5.76
S42	WIN_10	WIN_20	WIN_30	WIN_45	WIN_60	WIN_90	WIN_120	WIN_32	WIN_64
MAE	1.59	1.09	0.90	0.75	0.73	0.96	1.43	0.86	0.75
RMSE	1.81	1.22	1.01	0.84	0.79	1.00	1.58	0.94	0.80
S43	WIN_10	WIN_20	WIN_30	WIN_45	WIN_60	WIN_90	WIN_120	WIN_32	WIN_64
MAE	7.59	3.45	1.99	1.20	1.02	1.45	2.27	1.77	1.02
RMSE	7.92	4.03	2.46	1.44	1.34	1.88	2.65	2.21	1.38

S44	WIN_10	WIN_20	WIN_30	WIN_45	WIN_60	WIN_90	WIN_120	WIN_32	WIN_64
MAE	1.35	1.25	1.16	0.91	0.80	0.67	0.94	1.60	0.76
RMSE	1.99	2.02	1.83	1.35	1.18	1.08	1.07	2.91	1.14
S45	WIN_10	WIN_20	WIN_30	WIN_45	WIN_60	WIN_90	WIN_120	WIN_32	WIN_64
MAE	6.15	8.79	9.07	8.33	8.31	5.27	5.78	8.49	7.23
RMSE	7.55	9.62	9.76	9.34	9.26	7.75	6.57	9.35	8.61
S46	WIN_10	WIN_20	WIN_30	WIN_45	WIN_60	WIN_90	WIN_120	WIN_32	WIN_64
MAE	3.99	4.24	4.32	4.24	4.06	4.16	4.56	4.33	4.05
RMSE	5.22	5.29	5.27	5.05	4.63	4.49	4.69	5.26	4.57
S47	WIN_10	WIN_20	WIN_30	WIN_45	WIN_60	WIN_90	WIN_120	WIN_32	WIN_64
MAE	2.62	3.16	3.38	3.57	3.84	4.37	5.30	3.46	3.94
RMSE	2.94	3.27	3.43	3.61	3.87	4.48	5.53	3.50	3.97
S48	WIN_10	WIN_20	WIN_30	WIN_45	WIN_60	WIN_90	WIN_120	WIN_32	WIN_64
MAE	2.67	2.81	2.81	2.79	2.80	3.11	3.44	2.82	2.87
RMSE	3.12	3.14	3.15	3.12	3.14	3.52	3.91	3.17	3.22
S49	WIN_10	WIN_20	WIN_30	WIN_45	WIN_60	WIN_90	WIN_120	WIN_32	WIN_64
MAE	9.21	7.05	4.82	2.70	2.25	1.63	1.84	4.28	1.73
RMSE	10.11	7.53	5.37	2.88	3.41	1.88	1.85	4.51	1.94
S50	WIN_10	WIN_20	WIN_30	WIN_45	WIN_60	WIN_90	WIN_120	WIN_32	WIN_64
MAE	12.10	10.30	8.66	7.65	7.01	6.04	4.79	8.72	6.83
RMSE	12.14	10.40	8.81	7.79	7.14	6.32	5.38	8.92	6.97
S51	WIN_10	WIN_20	WIN_30	WIN_45	WIN_60	WIN_90	WIN_120	WIN_32	WIN_64
MAE		7.51	9.64	8.35	7.54	6.11	3.88	9.39	7.30
RMSE		8.20	9.67	8.40	7.67	6.59	5.26	9.42	7.47
S52	WIN_10	WIN_20	WIN_30	WIN_45	WIN_60	WIN_90	WIN_120	WIN_32	WIN_64
MAE	4.91	5.16	5.28	5.37	5.41	5.52	5.73	5.31	5.43
RMSE	4.96	5.19	5.30	5.37	5.41	5.53	5.74	5.32	5.44
S53	WIN_10	WIN_20	WIN_30	WIN_45	WIN_60	WIN_90	WIN_120	WIN_32	WIN_64
MAE	2.88	2.10	1.77	1.83	1.96	2.02	2.48	1.77	2.03
RMSE	3.89	2.94	2.25	2.13	2.21	2.24	2.82	2.19	2.26
